# Semisynthetic Abietic and Dehydroabietic Acid Derivatives and Triptoquinone Epimers Interfere with LPS-Triggered Activation of Dendritic Cells

**DOI:** 10.3390/molecules27196684

**Published:** 2022-10-08

**Authors:** Jelver A. Sierra, Katherine Gilchrist, Jorge H. Tabares-Guevara, Liliana Betancur-Galvis, Jose R. Ramirez-Pineda, Miguel A. González-Cardenete

**Affiliations:** 1 Grupo Inmunomodulación, Universidad de Antioquia UdeA, Calle 70 No. 52-21, Medellín 050010, Colombia; 2 Grupo de Investigación Dermatológica, Universidad de Antioquia UdeA, Calle 70 No. 52-21, Medellín 050010, Colombia; 3 Instituto de Tecnología Química, Universitat Politècnica de València-Consejo Superior de Investigaciones Científicas, Avda. de los Naranjos s/n, 46022 Valencia, Spain

**Keywords:** abietane diterpenes, dehydroabietic acid, triptoquinone, cytokines, dendritic cells, surface co-stimulatory molecules

## Abstract

Abietic acid (AA), dehydroabietic acid (DHA) and triptoquinones (TQs) are bioactive abietane-type diterpenoids, which are present in many edible vegetables and medicinal herbs with health-promoting properties. Evidence suggests that beneficial effects of diterpenes operate, at least in part, through effects on cells in the immune system. Dendritic cells (DCs) are a key type of leukocyte involved in the initiation and regulation of the immune/inflammatory response and natural or synthetic compounds that modulate DC functions could be potential anti-inflammatory/immunomodulatory agents. Herein, we report the screening of 23 known semisynthetic AA and DHA derivatives, and TQs, synthesized previously by us, in a multi-analyte DC-based assay that detects inhibition of pro-inflammatory cytokine production. Based on the magnitude of the inhibitory effect observed and the number of cytokines inhibited, a variety of activities among compounds were observed, ranging from inactive/weak to very potent inhibitors. Structurally, either alcohol or methyl ester substituents on ring A along with the introduction of aromaticity and oxidation in ring C in the abietane skeleton were found in compounds with higher inhibitory properties. Two DHA derivatives and two TQs exhibited a significant inhibition in all pro-inflammatory cytokines tested and were further investigated. The results confirmed their ability to inhibit, dose dependently, LPS-stimulated expression of the co-stimulatory molecules CD40 and/or CD86 and the production of the pro-inflammatory cytokines IL-1β, IL-6, IL-12 and TNFα. Our results demonstrate that DC maturation process can be targeted by semisynthetic DHA derivatives and TQ epimers and indicate the potential of these compounds as optimizable anti-inflammatory/immunomodulatory agents.

## 1. Introduction

Diterpenes are a heterogeneous group of naturally occurring compounds widely distributed in nature. Many diterpenes are abundant in edible vegetables and medicinal herbs, where they apparently contribute to their health-promoting effects. Pharmacological interest in diterpenes has increased as a consequence of the beneficial activities promoted by these compounds on the cardiovascular, nervous, respiratory and gastrointestinal systems in the context of several diseases [1,2,3]. Interestingly, diterpenes are prominent immunomodulatory compounds, as they have been shown to stimulate or inhibit important processes mediating the immune/inflammatory response. For instance, two representative diterpenes, such as Taxol and Phytol, and their derivatives, may exhibit immune stimulatory or immune inhibitory activity, in addition to their well-documented anti-tumor and antimicrobial properties [3,4]. Given the role played by the immune system in many diseases, studying the effects of diterpenes on immune cells and pathways could not only improve our understanding of the pleiotropic effects of these molecules, but also amplify and redefine their pharmacological applications. 

Dendritic cells (DCs) are a class of leucocytes that play an important role in innate and adaptive immunity [5]. DCs are present in peripheral and lymphoid tissues in an “inactivated” immature form, where they acquire self-antigens or microbial antigens. On the one hand, under non-inflammatory conditions, DCs promote immunological tolerance by destroying self-reactive T cells or by transforming them into anergic or regulatory T cells. On the other hand, the presence of infectious or aseptic inflammatory signals triggers a developmental process called maturation, characterized by the increased migration from periphery to draining lymph nodes where T cells are abundant, leading to their activation and differentiation into effector and memory T cells and the subsequent generation of long-lasting immunity. DC maturation is phenotypically defined as an increased expression of molecules related to antigen presentation (such as MHC class II and co-stimulatory molecules) on the DC surface and the abundant secretion of pro-inflammatory cytokines (such as IL-1, IL-6, IL-12 and TNF-α) [6]. It is known that the inflammatory environment generated during chronic inflammatory diseases, such as lupus, rheumatoid arthritis, atherosclerosis, ulcerative colitis, multiple sclerosis, psoriasis or obesity, alters DCs’ capacities to promote tolerance and that restoration of this function in DCs has a therapeutic impact on the disease [7]. This “pathogenic” to “tolerogenic” transition of DCs is usually associated with inhibition of maturation, as determined by reduced co-stimulatory molecule expression, pro-inflammatory cytokine secretion or both [7]. These observations, together with the realization that most of the commercial anti-inflammatory/immunosuppressive drugs are potent inhibitors of DC maturation [8], constitute the rationale for the search of novel natural or synthetic products endowed with an inhibitory effect on DC. 

**Figure 1:**
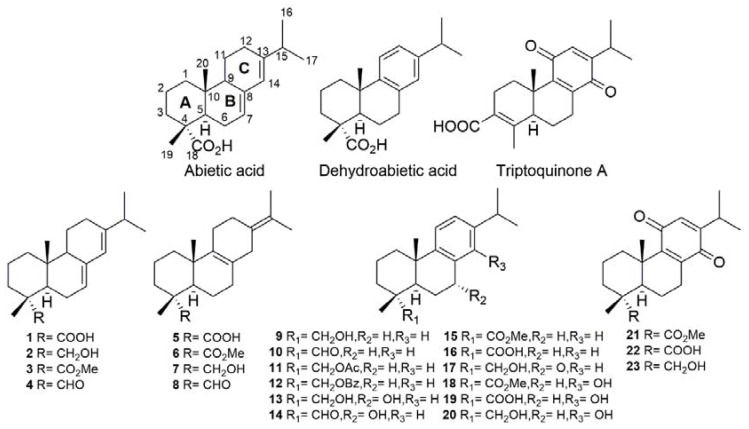
Bioactive and tested abietanes: abietanes **1**–**8**, dehydroabietanes **9**–**20** and triptoquinones **21**–**23**.

As a result of our continued search and design of bioactive terpenoids, we synthesized, from (−)-abietic acid, several abietic acid (AA) derivatives (**1**–**8**) [9], dehydroabietic acid (DHA) derivatives (**9**–**17**) [10] and dehydroabietanes **18**–**20** [11], as well as three triptoquinone C-4 epimers (**21**–**23**) [11] (TQs; Figure 1), and tested some biological activities. Interestingly, two of the semisynthetic TQ epimers inhibited the production of two important pro-inflammatory cytokines (IL-1β and TNF-α) in human LPS-stimulated monocytes [11], suggesting a potential anti-inflammatory activity on a DC precursor. 

Although the response of DCs to diterpenes and diterpene-containing natural products has been reported, to the best of our knowledge, the effects of AA/DHA derivatives and triptoquinones on this cell type are unknown. A remarkable exception is the related compound triptolide, a diterpene epoxide responsible for the multiple immunological activities of the Chinese medicinal herb *Tripterygium wilfordii*, for which an inhibitory effect of mouse and human DC maturation has been reported [12,13,14,15]. This fact indicates a promising DC modulatory potential in this group of molecules. Herein, we report the screening of 23 known AA/DHA derivatives and three TQs, synthesized previously by us [9,10,11], in a robust DC-based assay that detects inhibition of pro-inflammatory cytokine production and the subsequent confirmation that at least four of them significantly inhibit DC maturation.

## 2. Results

### 2.1. Screening of 23 Semisynthetic Diterpenes in a DC-Based Bioassay 

Given the well-recognized roles of DCs in the normal functioning of the innate and adaptive immune systems and the association of excessive pro-inflammatory responses of DCs during chronic inflammation, the evaluation of the effects of natural and synthetic compounds on the inflammatory DC response is of great interest as a read out for the search of anti-inflammatory/immunomodulatory compounds. Thus, we began the present work by screening 23 semisynthetic diterpenes (AA derivatives **1**–**8**, DHA derivatives **9**–**20** and TQs **21**–**23**; see Figure 1 for molecular structures) by using a multi-analyte Luminex-based assay. The assay measures the concentration of the cytokines IL-1β, IL-6, IL-12p40, IL-12p70 and TNF-α, released to the supernatants by mouse primary DC cultures in response to LPS stimulation, as we previously reported with other natural products [16]. As expected, control untreated DC or vehicle (DMSO 0.25%)-treated DC produced very low or non-detectable levels of the tested pro-inflammatory cytokines, and Dexamethasone (Dexa), a known DC inhibitor, blunted cytokine levels in those resting DCs (Table 1). LPS (0.1 μg/mL, see Methodology for details), a powerful microbial DC activator, induced profuse amounts of all tested cytokines, which, in turn, were substantially inhibited (66–98%) when the cultures also contained Dexa (Table 1). Once the biosensor DC system and controls were working appropriately, we continued by testing the semisynthetic diterpenoids. In prior experiments, we did not observe a cytotoxic effect or cytokine-inducing activities for any of the semisynthetic compounds in resting DCs at the concentration tested during the screening (10 μg/mL, ~30 μM; data not shown). Interestingly, when we pre-treated DCs for 6 h with the abietane diterpenoids and then stimulated them with LPS for 18 additional hours, we observed a variety of responses, depending on the compound and the cytokine (Table 1). All compounds were inhibitory, to some degree, for at least two cytokines. In general, the pattern exhibited is that TNF-α and IL-1β was inhibited to some degree by all compounds, whereas the other cytokines (IL-12 and IL-6) tended to be less inhibited and, in some cases, were even stimulated in response to compounds. The magnitude of the inhibitory effect and the number of cytokines inhibited was, in general, higher for DHA derivatives and TQs than for AA derivatives. To establish whether a particular level of inhibition was significant, we defined the cut-off as the arithmetic mean-2SD of the control DC cultures (vehicle- and LPS-treated DC, *n* = 28) and marked those compounds that inhibited cytokine production below the cut-off level. This analysis indicated, again, that DHA derivatives and TQs were more potent than AA derivatives on the basis of the number of cytokines significantly inhibited. Among the eight AA derivatives, two were ineffective, three inhibited only one cytokine and three inhibited two or more cytokines. Interestingly, from 15 DHA derivatives and TQs, only 1 was ineffective and the remaining 14 compounds inhibited 2 or more cytokines (Table 1). Remarkably, DHA derivative **17** and TQs **21** and **23** were extremely powerful, as they inhibited TNF-α production by more than 90% and DHA derivatives **9** and **18** and TQs **21** and **23** significantly inhibited all cytokines tested (Table 1). Among all, TQs **21** and **23** appear to be the most potent compounds.

### 2.2. Structure–Activity Analysis

**Scheme 1:**
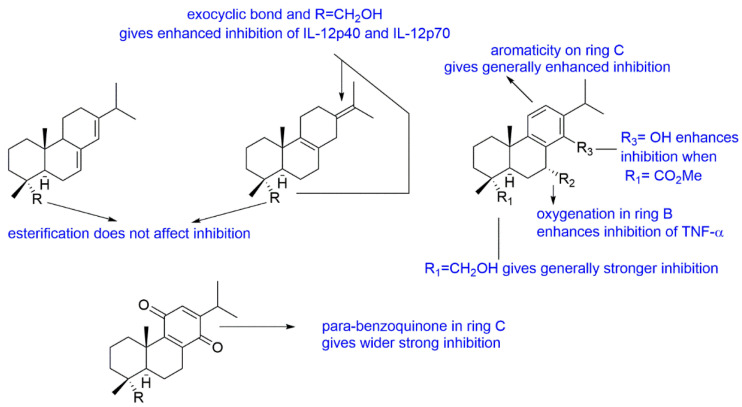
Main structure–activity relationships after screening of abietane-based compounds **1**–**23**.

By comparing the activities of the tested compounds in terms of chemical structure, we could obtain some interesting structure–activity relationships (Scheme 1). As it can be noticed in Table 1, compounds **1**–**8** lacking an aromatic ring C did not generally suppress the secretion of the pool of pro-inflammatory cytokines, displaying some effect on the secretion of TNF-α and IL-1β. Only compounds **1** (abietic acid, AA), methyl abietate (**3**, MA) and isomerized abietanol **7** surpassed the cut-off values. Compound **3** displayed an identical profile to AA, suggesting that methyl esterification does not affect the activity; however, we could not rule out a reduction in the cytokine levels due to cytotoxicity of AA, which accounted for about 35%. The effect of compound **7** was stronger in restraining the activation of DC than AA (**1**) and, although it did not inhibit the secretion of IL-6, its remarkable ability to inhibit secretion of IL-12(p40) and IL-12p70 should be given further attention. Roughly, the introduction of aromaticity in ring C of the abietane skeleton results in the dehydroabietic acid and derivatives, compounds **9**–**20**, which, compared to the AA derivatives family, displayed an increased ability to inhibit the secretion of pro-inflammatory cytokines TNF-α, IL-1β and IL-12p70, excluding compounds **12**, **15** and **19**. Even more, from all dehydroabietanes, compounds **9**, **17** and **18**, in addition to the cytokines already mentioned, also suppressed the secretion of IL-12(p40) and IL-6. Among the dehydroabietanes **9**–**20**, those possessing either a methyl ester or an alcohol group at C18 and an oxidized C ring were the most potent inhibitors. It is also of interest that oxygenation at ring B, compounds **13** and **17**, gave enhanced inhibition towards TNF-α. Finally, the unsaturation together along benzoquinone moiety seen in TQs **21** and **23** lead to the most suppressing compounds on the cytokine secretion profile of DC. This did not happen with compound **22**, which displays a more polar substitution on ring A as carboxylic acid (this seems to be also true for compounds **1**, **5**, **16** and **19**).

### 2.3. Selected DHA Derivatives and TQs Inhibit DC Maturation in a Dose-Dependent Manner

Since our screening assay evidenced a number of compounds with a potent inhibitory effect on LPS-induced cytokine production by DCs (Table 1), we continued the study with those exhibiting a significant inhibition of all the tested cytokines, as they would represent potential leads with a more integral inhibition of the inflammatory pathways triggered by LPS. Thus, we used different concentrations of compounds **9**, **17**, **18**, **21** and **23** to treat DC cultures following the described protocol, but in this case, we collected and used cells to assess the surface expression of the co-stimulatory molecules CD40 and CD86 by flow cytometry. CD40 and CD86 are not only essential molecules for the critical DC function of antigen presentation to T cells, but also, a high level of expression of these markers on the DC surface is a common indicator of DC maturation. Compounds able to inhibit the expression of surface co-stimulatory molecules are expected to potentially inhibit inflammatory response and promote tolerance [8]. In a previous report, we showed that, on day 9 of differentiation, primary bone marrow-derived DC-expressed low levels of MHC-II and co-stimulatory molecules CD40 and CD86 on the surface and stimulation with LPS (3 µg/mL) resulted in the up-regulation of the those markers [17]. Here, again, we observed that LPS treatment (0.1 μg/mL) also increased the frequency of CD40+ and CD86+ DCs and the surface density of the marker, as evidenced by a higher mean of fluorescent intensity (MFI) (Table 2). Treatment with Dexa prior to LPS stimulation reduced the MFI and/or the percentage of CD40+ and CD86+ DC, as expected, indicating a functional impairment of the maturation process (Table 2) and validating the system to test the effect of abietane diterpenoids. Based on the basal cytotoxicity of several hydroxyabietanes and triptoquinone epimers on the Jurkat and HeLa cell lines [9,10,11] and the results of the screening reported here (Table 1), we evaluated abietane diterpenoids using semi log-spaced concentrations of 1.0, 3.2 and 10.0 μg/mL for these experiments. All the concentrations tested for all compounds were subtoxic and revealed, in general, a dose-dependent inhibition of LPS-stimulated DC maturation, with higher doses exhibiting stronger effects (Table 2). Compounds **9** and **23** significantly down-regulated the expression of CD40 and showed a milder effect on CD86, whereas TQ **21** displayed the opposite trend, inhibition of CD86 with mild effects on CD40. Notably, DHA derivative **18** significantly inhibited the surface expression of both markers (Table 2) and was the most potent synthetic diterpenoid to inhibit co-stimulatory molecule expression. 

**Figure 2:**
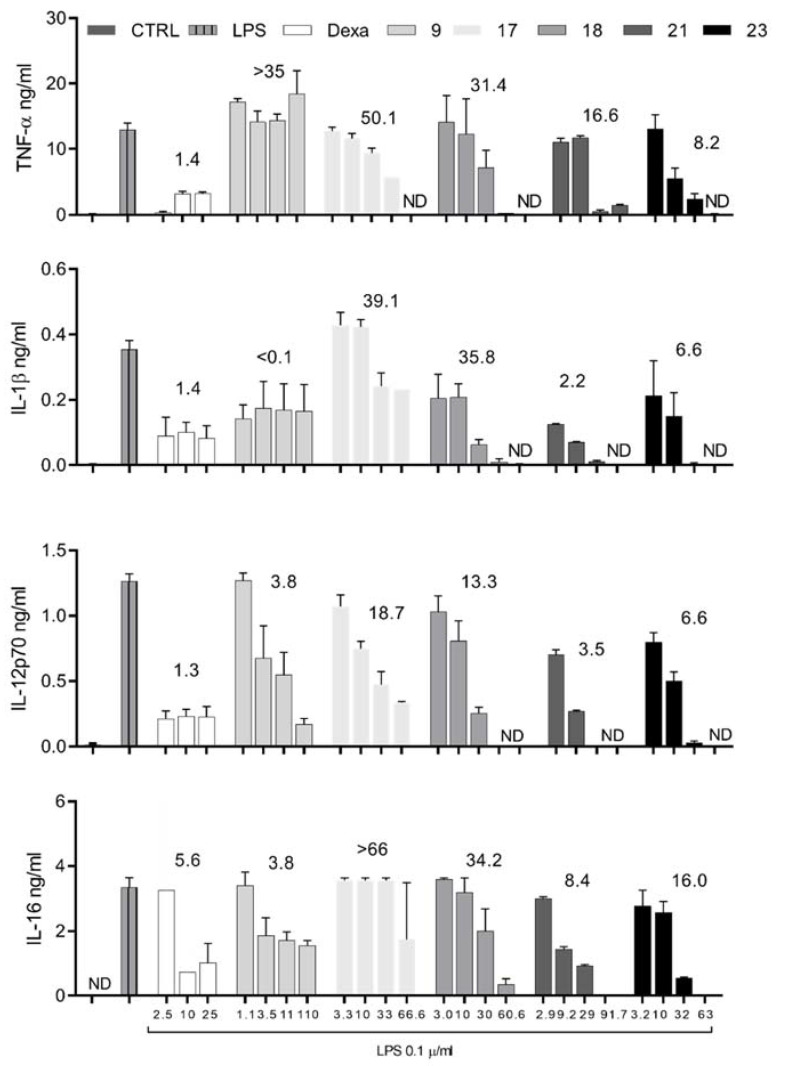
Dose-dependent inhibition of pro-inflammatory cytokine production in DCs by selected semisynthetic abietane diterpenoids. Dendritic cells were generated and treated with the indicated concentration of DHA derivatives **9**, **17** and **18** and TQs **21** and **23** and subsequently stimulated with LPS and the concentration of cytokines released to supernatants was determined by ELISA assay (see Methodology for details). Dexa was used as a positive control whereas vehicle-treated LPS-stimulated DCs were used as negative control. A control with untreated DCs is also shown. Results are reported as the mean ± SEM of two independent experiments. Numbers above each bars group stands for IC_50_ values. ND Non detectable.

Finally, given the availability of supernatants from these experiments, we took advantage by quantifying the concentration of the cytokines IL-1β, IL-6, IL-12p70 and TNF-α by ELISA assay. The production of the pro-inflammatory cytokines was almost abolished (TNF-α, IL-1β, IL-6and IL-12p70) by our control drug Dexa (Figure 2), as expected. Results with abietane diterpenoids were in fair agreement with the screening assay and additionally revealed the general trend of selected compounds **9**, **17, 18**, **21** and **23** to inhibit pro-inflammatory cytokine production in a dose-dependent manner (Figure 2). This assay pointed again towards TQs **21** and **23** as the most potent inhibitors of pro-inflammatory cytokine production in DC.

## 3. Discussion

The increasingly recognized role of DCs to instruct/promote either a pathogenic or a protective T cell immune response explains the increasing interest from the scientific community to find molecules that alter DC physiology. Many natural or synthetic agents that stimulate or inhibit DC activities have been reported as recognized or potential immunotherapeutical or immunoprophylactic agents [8,18,19]. Since fully mature DCs that express high levels of pro-inflammatory cytokines and co-stimulatory molecules are drivers of inflammatory response, compounds that target mature DCs can be proposed as potential anti-inflammatory agents. Here, we report, as result of a DC bioscreening, four semisynthetic abietane derivatives that significantly inhibit DC maturation and, therefore, represent potential leads for the development of anti-inflammatory or immunomodulatory agents for the treatment of inflammatory diseases. 

We used TLR4 triggering with the prototypical stimulus LPS as a DC maturation inducer to screen 23 semisynthetic abietane-derivatives. TLR4 transduce exogenous and endogenous inflammatory signals via signaling pathways, such as NF-κB and MAPK/AP1, to promote the production of soluble inflammatory mediators (such as cytokines, chemokines, arachidonic acid-derived mediators, nitric oxide, etc.) and cell receptors (such as co-stimulatory molecules and MHC class II) [20,21]. TLR4 activation secondarily triggers other inflammatory pathways, such as JAK/STAT, via a secreted cytokine and cytokine receptor and, together, all these pathways are involved in the pathogenesis of many inflammatory diseases [22]. From the 23 abietane derivatives, at least five (compounds **9**, **17**, **18**, **21** and **23**) were consistent inhibitors of DC maturation, as assessed by surface CD40 and CD86 expression and IL-1β, IL-6, IL-12p40, IL-12p70 and TNF-α secretion. To the best of our knowledge, this is the first report of a mature DC-inhibitory activity for these compounds and is in agreement with that observed in related diterpenes with this cell type [12,13,14,15,23]. Results would also extend to DCs observed in LPS-activated macrophages, in which inhibition of either pro-inflammatory cytokines or nitric oxide secretion was achieved after treatment with diterpenoids sharing the abietane carbon skeleton, such as AA/DHA [24,25], ferruginol and 18-methylester ferruginol [26,27], hinokiol [26], callistrisic, majusanic and angustanoic acids [28], carnosol and carnosic acid [29,30]. Although the particular signaling pathways mediating the DC maturation inhibitory effect reported here remain to be investigated, the reported inhibition of NF-κB by tetrahydroabietic acid [24] and of NF-κB and JAK/STAT pathways by carnosol and carnosic acid [31,32] suggest a potential mechanism. Those studies and our report support the idea that abietane diterpenoids exhibit a multitude of biological activities [33], at least in part, through immune modulation and that these compounds may target DCs in addition to macrophages and other leucocytes.

Structure–activity relationships (SARs) are fundamental to several aspects of drug discovery, ranging from primary screening to lead optimization [34]. In our screening assay, AA derivatives were less active than DHA and TQs. In particular, compound **23** is the C-4 epimer of natural triptoquinone D, which has shown potent inhibitory activities against IL-lα and IL-1β, releases for human peripheral mononuclear cells [35]. Thus, it can be concluded that stereochemistry at C-4 is less important to determine the principal activity, which is probably due to the benzoquinone moiety, as happens with natural triptoquinone A (Figure 1), another known interleukin-1 inhibitor [36]. Overall, data suggest that the anti-inflammatory activity of abietane diterpenes seems to rely mostly on ring C, as alterations in DC biology have been described for related and already known anti-inflammatory compounds with oxidized C rings i.e., triptolide [14,15] and carnosol [23].

In the immune system, DCs are considered the sensors that translate environmental signals to the adaptive immune system, directing the type of adaptive immune response required. For example, when DCs produce IL-12 in response to a particular stimulus, a Th1 type of immune response is triggered in the CD4+ T cell compartment. Although we did not perform experiments to evaluate antigen presentation to CD4+ T cells by abietane-conditioned DCs, the observation that several semisynthetic abietanes inhibit IL-12p70 secretion suggests the potential of these chemical leads for diseases where an aberrant proinflammatory Th1 CD4+ T cell response is present, such as autoimmune and other chronic inflammatory diseases. In this sense, compounds **6** or **7**, which inhibited two forms (p70 and p40) of the IL-12 cytokine family in a selective manner (little or no inhibition of other cytokines, Table 1), might be leads of particular interest that deserve further attention. Other compounds inhibiting IL-12 were less selective as they also inhibited other pro-inflammatory cytokines (Table 1) and could be potential leads to inhibit several forms of inflammatory responses besides Th1. Co-stimulatory surface molecules are also key signals that, together with cytokines, contribute to T cell expansion and differentiation. Thus, the observation that the four most potent abietanes described here exhibit a particular pattern for CD40, CD86 and pro-inflammatory cytokine inhibition (Table 2 and Figure 2) implies that abietane-derived semisynthetic compounds could be exploited in the future for a more rational and tailored immune inhibition.

## 4. Materials and Methods

### 4.1. Chemicals and Reagents

Compounds were prepared from commercially available abietic acid (Aldrich, Saint Louis, USA) following reported literature procedures [9,10,11]. Stock solutions of compounds were prepared in anhydrous dimethylsulfoxide (DMSO) and kept frozen at −70 °C until required. The concentration of DMSO in biological assays was under 0.25%.

### 4.2. Mice and Bone Marrow Cell Preparation

C57BL/6 mice, originally obtained from Charles River Laboratories (Wilmington, MA, USA), were bred and housed under barrier conditions at the SPF animal facility of the Sede de Investigaciones Universitaria (SIU), Universidad de Antioquia. The institutional ethical committee approved all procedures involving animals. Periodically serologic and microbiological tests performed on the mice colony confirmed the absence of murine pathogens. Total bone marrow cells were collected from aseptically removed femurs and tibiae of 8–12-week-old female mice. The bone marrow cell content was flushed with the aid of a tuberculin syringe obtaining a homogeneous cell suspension.

### 4.3. Dendritic Cell (DC) Generation

DCs were generated from bone marrow precursors according to the method reported by Lutz et al. (1999) [37] with minor modifications. Thus, 2 × 10^5^ bone marrow cells/mL were washed and cultured in RPMI 1640 (Glutamax, GIBCO, New York, NY, USA) supplemented with 10 mM HEPES, 2 mM L-glutamine, 10% FBS, 50 μM β-mercaptoethanol and recombinant murine granulocyte/macrophage colony-stimulating factor (200 U/mL, rmGM-CSF). Cultures were maintained under standard culture conditions in 5% CO_2_ atmosphere at 37 °C. On days 3 and 6 of culture, fresh medium containing rmGM-CSF (200 U/mL) was added. Non-adherent cells were collected on day 9, washed and suspended at 1 × 10^6^ cells/mL in fully supplemented culture medium for further use. These cells were morphologically, phenotypically (CD11c^+^ CD11b^+^ MHC class II^+^ Gr1^−^ CD4^−^ CD8^−^ CD19^−^) and functionally (potent APC in allogeneic mixed leucocyte reactions) consistent with DC_S_ (data not shown).

### 4.4. Cell Treatments

For the screening assay 100 µL of a DC suspension 1 × 10^6^ cells/mL was seeded in 96-well plates and cultured in the presence of 10 μg/mL of semisynthetic abietane derivatives (~30 μM). Vehicle (0.25% DMSO) or Dexamethasone (Alexis Biochemicals, San Diego, USA; also prepared in DMSO as vehicle) -treated DCs were used as negative and positive controls, respectively. After 6 h of incubation, cells were stimulated with LPS (0.1 μg/mL, Sigma, Saint Louis, MO, USA) for additional 18 h. DC cultures incubated in the absence of LPS were also set as non-stimulated controls. Cell-free supernatants were finally collected for further assessment of cell viability and cytokine secretion by Luminex technology. For the dose-response experiments, DCs (1 × 10^6^ cells/mL/well) were plated onto 24-well plates, then pre-treated with semisynthetic abietane derivatives or control reagents for 6 h and stimulated with 0.1 μg/mL of LPS for additional 18 h. Cells were collected by scrapping, washed and re-suspended in PBS to subsequently analyze surface costimulatory molecules, whereas supernatants were stored at −20 °C for further cytokine quantifications by ELISA assay.

### 4.5. Cell Viability, Co-Stimulatory Molecule Expression and Cytokine Secretion

Cell viability was determined by measuring the activity of the enzyme lactate dehydrogenase released from the cytosol of DC to the supernatant using the Cytotox 96^®^ Non-radioactive cytotoxicity assay kit (Promega, Madison, WI, USA), following manufacturer’s protocol. The percentage of viable cells was calculated with the formula: % viability =100 − [Experimental LDH release (OD490)/Maximum LDH release (OD490) × 100]. As a surface marker indicator of DC maturation, the abundancy of the co-stimulatory molecules CD40 and CD86 was determined by flow cytometry. Cell staining was performed using a combination of FITC-conjugated anti-CD40 and PE-conjugated anti-CD86 and PE-Cy7-conjugated CD11c antibodies after a blocking step with FBS and purified rat anti-mouse CD16/CD32 (clone2.4G2) at 4 °C for 10 min. Appropriate isotype-matched control antibodies were used. Optimal dilutions of all antibodies were established in preliminary experiments. Cells were analyzed using an FACSCanto flow cytometer (BD Biosciences, San Jose, CA, USA) and data were analyzed using WinMdi software (Scripps, La Jolla, CA, USA). Forward and side scatter parameters were used to gate live cells. Results were reported as the percentage of CD40 and CD86-positive cells and the mean of fluorescent intensity (MFI) as an indicator of the surface marker density. Supernatants from the screening assay were used to quantify the levels of the cytokines IL-1β, IL-6, IL-12p40, IL-12p70 and TNF-α using the Luminex technology with a customized multiplexed kit (Bioplex multiplex mouse Cytokine Assay kit, Bio-Rad, Hercules, CA, USA) according to the manufacturer’s protocol. The limits of detection of this method were 9.4, 0.2, 2.3, 1.4 and 0.4 pg/mL for IL-1β, IL-6, IL-12p70, TNF-α and IL-12p40, respectively. Data for cytokine arrays were collected by using the Bio-Plex suspension array system and analyzed with the Bio-Plex Manager™ (Bio-Rad, Hercules, CA, USA). In the supernatants from the dose–response experiments, the cytokine concentrations (IL-1β, IL-6, IL-12p70 and TNF-α) were determined by ELISA following manufacturer’s protocols (BD Bioscience). Detection thresholds were 3, 15.6, 62.5 and 1.07 pg/mL, for IL-1β, IL-6, IL-12p70 and TNF-α, respectively.

### 4.6. Data Analysis

For the screening assay, the concentration of cytokines in the supernatants of cells treated with abietane derivatives was reported, as well as the percentage of inhibition (based on the concentration of the vehicle-treated and LPS-stimulated control). Those compounds that inhibited cytokine production below the cut-off level (mean-2SD of the LPS-stimulated control, based on more than 20 replicate wells collected from more than 7 experiments) were considered significant inhibitors. For the dose–response experiments, results (cytokine concentration, percentage of CD40+ and CD86+ cells and MFI) were reported as the mean ± SEM. Data collection and analysis, as well as ANOVA tests performed using the GraphPad PRISM 7.0 software (GraphPad Software Inc., San Diego, CA, USA).

## 5. Conclusions

In summary, this report shows the potential of some abietane-containing molecules to target DC maturation, increasing the interest in these compounds for immune modulation and suggesting that many abietane-containing products promote beneficial health effects via DCs and downstream responses. Future optimization and a better understanding of the mechanisms of action of abietanes will be key for a future therapeutic exploitation.

## Figures and Tables

**Table 1:** Screening for the cytokine-inhibiting activity of 23 semisynthetic abietane diterpenes at 10 μg/mL.

Compound	Concentration µM	Cytokine Concentration in ng/mL (% Inhibition)					Cell Viability
		TNF-α	IL-1β	IL-12p70	IL-12p40	IL-6	
**Control**		ND	0.06 ± 0.04	0.01 ± 0.013	0.27 ± 0.08	0.03 ± 0.05	100.4
**Vehicle**		0.01 ± 0.01	0.08 ± 0.06	0.01 ± 0.06	0.38 ± 0.05	0.01 ± 0.01	95.7
**Dexa**		ND	0.02 ± 0.01	ND	0.06 ± 0.01	ND	98.7
**LPS**		3.2 ± 0.61	2.5 ± 0.36	1.00 ± 0.12	28.79 ± 5.67	15.36 ± 3.13	99.3
**1**	33.1	**1.48 (53.8)**	1.54 (38.4)	0.84 (16.0)	22.52 (21.8)	11.03 (28.2)	65.0
**2**	34.7	2.26 (29.4)	1.61 (35.6)	1.08 (−8.0)	26.89 (6.6)	13.46 (12.4)	92.2
**3**	33.1	**1.46 (54.4)**	1.63 (34.8)	0.94 (6.0)	27.47 (4.6)	11.55 (24.8)	100.5
**4**	34.9	2.07 (35.3)	2.12 (15.2)	1.37 (−37.0)	31.04 (−7.8)	19.39 (−26.2)	103.4
**5**	34.7	2.48 (22.5)	2.25 (10.0)	1.02 (−2.0)	37.96 (−31.9)	13.81 (10.1)	103.0
**6**	33.1	2.61 (18.4)	1.93 (22.8)	0.82 (18.0)	17.40 (39.6)	13.27 (13.6)	104.5
**7**	34.7	1.99 (37.8)	**0.47 (81.2)**	**0.14 (86.0)**	**4.79 (83.4)**	16.16 (−5.2)	101.0
**8**	34.9	2.09 (34.7)	1.70 (32.0)	1.05 (−5.0)	29.01 (−0.8)	27.93 (−81.8)	104.3
**9**	34.9	**0.66 (79.4)**	**0.62 (75.2)**	**0.34 (66.0)**	**12.65 (56.1)**	**7.53 (51.0)**	94.4
**10**	35.2	**1.29 (59.8)**	**0.94 (62.4)**	0.53 (47.0)	15.04 (47.8)	11.85 (22.9)	102.5
**11**	30.4	**1.34 (58.0)**	**1.17 (53.2)**	0.56 (44.0)	23.5 (18.2)	12.85 (16.3)	102.5
**12**	25.5	2.26 (29.5)	1.94 (22.4)	1.23 (−23.0)	21.53 (25.2)	22.15 (−44.2)	98.9
**13**	34.7	**0.47 (85.3)**	**0.95 (62.0)**	**0.46 (54.0)**	28.81 (−0.1)	11.83 (23.0)	97.0
**14**	33.3	**0.69 (78.5)**	1.37 (45.2)	**0.49 (51.0)**	22.97 (20.2)	11.36 (26.0)	92.5
**15**	31.8	2.79 (12.8)	**1.16 (53.6)**	1.16 (−16.0)	15.61 (45.8)	17.60 (−14.6)	103.6
**16**	33.3	**0.64 (80.1)**	**0.96 (61.6)**	0.53 (47.0)	24.40 (15.2)	15.71 (−2.3)	106.2
**17**	33.3	**0.27 (91.7)**	**0.41 (83.6)**	**0.39 (61.0)**	15.25 (47.0)	10.10 (34.2)	98.0
**18**	30.3	**0.48 (85.1)**	**0.40 (84.0)**	**0.39 (61.0)**	15.29 (46.9)	**7.61 (50.5)**	96.1
**19**	31.6	**1.60 (50.0)**	1.50 (40.0)	0.84 (16.0)	30.26 (−5.1)	11.92 (22.4)	100.7
**20**	33.1	**0.75 (76.6)**	**0.76 (69.6)**	**0.42 (58.0)**	28.52 (0.9)	9.70 (36.8)	97.7
**21**	29.0	**0.15 (95.3)**	**0.40 (84.0)**	**0.13 (87.0)**	**8.61 (70.1)**	**3.64 (76.3)**	101.5
**22**	30.3	**1.49 (53.4)**	1.54 (38.4)	0.84 (16.0)	32.0 (−11.1)	11.00 (28.4)	103.2
**23**	31.6	**0.12 (96.2)**	**0.61 (75.6)**	**0.25 (75.0)**	**10.13 (64.8)**	**6.41 (58.3)**	98.5
**Dexa/LPS**	10.0	0.08 ± 0.03	0.32 ± 0.03	0.25 ± 0.06	5.74 ± 3.89	5.21 ± 1.67	98.5

Results from control (untreated), vehicle- (DMSO 0.25%), Dexa (10 µM), LPS- (DMSO 0.25% + LPS 0.1 µg/mL) or Dexa/LPS-treated DC are also shown as the mean ± SEM. Compounds **1**–**23** were tested at 10 µg/mL in LPS-stimulated DC. Cell viability after each treatment is also shown. Magnitude of the inhibitory effect >50% is indicated in bold. Cut-off levels were 1.98, 1.78, 0.76, 17.45 and 9.1 ng/mL for TNF-α, IL-1β, IL-12p70, IL-12p40 and IL-6, respectively. Underlined are those compounds that exhibited significant inhibition for all cytokines tested. ND Non detectable.

**Table 2:** Effect of selected semisynthetic abietane diterpenoids on co-stimulatory molecule expression in DC.

Compound	Concentration in μM	CD40 MFI (% CD40+ Cells)	CD86 MFI (% CD86+ Cells)	Cell Viability
Control	-	54.7 ± 9.5 (16.4 ± 3.0)	45.7 ± 5.0 (11.3 ± 1.5)	99.31 ± 0.7
LPS	-	267.6 ± 5.5 (62.8± 2.6)	104.7 ± 2.4 (53.7 ± 3.0)	100.9 ± 0.5
LPS + 9	0.11	264.7 ± 13.3 (64.6 ± 0.7)	98.4 ± 2.8 (55.1 ± 0.2)	100.9 ± 1.7
	3.49	248.0 ± 10.4 (63.7 ± 1.7)	122.4 ± 7.3 (54.5 ± 1.5)	102.7 ± 2.4
	11.03	260.9 ± 29.0 (66.6 ± 0.3)	129.9 ± 8.3 (55.7 ± 1.1)	98.5 ± 1.7
	34.9	151.1 ± 1.3 ^a^ (69.3 ± 6.7)	106.4 ± 9.9 (44.3 ± 1.4)	84.9 ± 11.3
	110.3	NA	NA	4.5 ± 2.7
LPS + 17	3.3	148.8 ± 49.0 (78.0 ± 3.0)	100.8 ± 20.0 (53.6 ± 9.3)	104.6 ± 0.1
	10.5	179.5 ± 57.3 (72.7 ± 7.4)	99.1 ± 23.4 (44.7 ± 10.3)	102.6 ± 0.4
	33.3	161.9 ± 36.7 (71.3 ± 7.3)	95.0± 31.1 (45.1 ± 10.2)	98.0 ± 0.8
	66.6	32.3 ± 21.7 ^a^ (32.3 ± 21.7)	65.5 ± 51.1 (34.0 ± 16.1 ^c^)	84.0 ± 7.8
LPS + 18	3.03	164.9 ± 68.9 ^a^ (48.9 ± 11.7)	96.0 ± 22.8 (42.9 ± 8.1)	98.4 ± 3.3
	9.6	122.8 ± 34.7 ^a^ (50.9 ± 10.3)	65.8 ± 22.9 ^b^ (32.0 ± 9.4 ^c^)	99.0 ± 3.2
	30.3	110.4 ± 30.7 ^a^ (45.1 ± 9.9)	65.2 ± 21.9 ^b^ (26.0 ± 7.3 ^c^)	95.3 ± 2.4
	60.6	17.3 ± 3.8 ^a^ (11.0 ± 4.4)	19.9 ± 7.7 ^a^ (6.9 ± 3.2 ^a^)	81.0 ± 1.6
	95.6	NA	NA	47.5 ± 1.4
LPS +21	2.9	216.6 ± 4.1 (39.7 ± 7.6)	88.2 ± 1.4 (38.3 ± 0.9)	103.5 ± 0.4
	9.2	263.6 ± 0.4 (46.2 ± 8.9)	72.1 ± 0.5 ^c^ (30.2 ± 2.4 ^c^)	102.4 ± 1.2
	29.0	170.6 ± 0.1 ^a^ (38.1 ± 7.5)	22.9 ± 0.4 ^a^ (30.7 ± 1.5 ^c^)	100.8 ± 1.8
	91.7	0.1 ± 0.1 ^a^ (0.05 ±0.05 ^a^)	0.1 ± 0.1 ^a^ (16.7 ± 0.7 ^a^)	85.5 ± 1.4
LPS +23	3.2	157.3 ± 34.0 ^a^ (71.0 ± 4.3)	120.0 ± 3.9 (53.7 ± 2.9)	100.4 ± 2.7
	10.0	161.2 ± 51.5 ^a^ (68.7 ± 5.8)	120.2 ± 3.0 (51.6 ± 3.1)	99.7 ± 1.9
	31.6	76.5 ± 7.3 ^a^ (57.0 ± 7.5)	82.2 ± 4.4 (38.5 ± 2.6)	93.2 ± 0.2
	63.2	39.7 ± 3.0 ^a^ (30.5 ± 3.5)	40.4 ± 11.6 ^a^ (33.4 ± 1.3 ^a^)	97.6 ± 6.1
LPS + Dexa	10.0	149.5 ± 9.2 ^a^ (66.0 ± 3.1)	119.5 ± 5.1 (30.9 ± 2.7 ^a^)	98.8 ± 0.6
	25.5	173.9 ± 10.1 ^a^ (61.3 ± 5.5)	89.4 ± 11.6 (25.9 ± 2.9 ^a^)	103.3 ± 1.1

Results from Control (untreated) DC (DMSO 0.25%,) or DC treated with vehicle + LPS (DMSO 0.25% + LPS 0.1 µg/mL), or LPS (0.1 µg/mL + Dexa) are also shown. Compounds **9**, **17**, **18**, **21** and **23** were dissolved in DMSO and tested at the indicated concentration in LPS-stimulated DC. The percentages of viable cells with respect to control untreated DC cultures are also shown. Results are presented as the mean ± SEM. Post hoc comparisons of groups against vehicle-treated LPS-stimulated DC in ANOVA are expressed as follow ^a^ *p* value < 0.001, ^b^ *p* value < 0.01, ^c^ *p* value < 0.05. NA non assessed.

## Data Availability

Not applicable.

## Sample Availability

Samples of the compounds are not available from the authors.
